# The effect of preventive use of hydrolyzed protein formula milk on gastrointestinal diseases and physical development of premature infants: A protocol for systematic review and meta-analysis

**DOI:** 10.1097/MD.0000000000023398

**Published:** 2020-11-20

**Authors:** Qiyan Yang, Qun Lin, Keni Chen, Juan Cao, Yonghong Feng, Shuli Han

**Affiliations:** a Department of Nursing, Hainan Modern Women and Children's Hospital, No.18-1, Qiongzhou Avenue, Fucheng Street; b Department of Neonatology, Haikou Hospital of the Maternal and Child Health, NO.6 Wentan Road, Guoxing Avenue; c Department of Pediatrics, Hainan Modern Women and Children's Hospital, No.18-1, Qiongzhou Avenue, Fucheng Street; d Department of Child Health Care, Haikou Hospital of the Maternal and Child Health, NO.6 Wentan Road, Guoxing Avenue, Qiongshan District, Haikou, Hainan, China.

**Keywords:** feeding intolerance, hydrolyzed protein formula milk, nutritional support, premature infants, standard preterm infant formula, systematic review and meta-analysis, very low birth weight infants

## Abstract

**Background:**

Because of the controversy in clinical nutritional support therapy of hydrolyzed protein formula milk and standard preterm infant formula (SPIF) in premature infants. In this study, the effectiveness and safety of preventive use of hydrolyzed protein formula milk in reducing gastrointestinal diseases and promoting physical development of premature infants are scientifically evaluated by systematic evaluation. To help find the suitable nutritional support for premature infants.

**Methods:**

To search the database of Chinese and English by computer: SinoMed, CNKI, WanFang Data, VIP, PubMed, EMbase and The Cochrane Library, and to collect randomized controlled trials on the application of hydrolyzed protein formula milk in nutritional support treatment of premature infants compared with SPIF. The retrieval time limit is from the establishment of each database to September 1, 2020. Two authors independently completed the paper search, and sorting out the main outcome indicator and secondary outcome indicator in the selected literature, and the data are statistically analyzed by Review Manager software (RevManV.5.3.0) and STATA 13.0.

**Results:**

This study will provide a high-quality evidence on the effects of hydrolyzed protein formula milk on gastrointestinal diseases and physical development of premature infants.

**Conclusion:**

At present, the clinicians are controversial about the safety and effectiveness of hydrolyzed protein formula milk and SPIF in the nutritional support therapy of premature infants. This study will compare the effectiveness and safety of these 2 nutritional support methods, and make a comprehensive analysis of the influence of hydrolyzed protein formula milk on gastrointestinal diseases and physical development of premature infants, and finally give a positive conclusion.

**OSF registration number:**

DOI 10.17605/OSF.IO/UQD92

## 1 Introduction

With the gradual improvement of neonatal intensive care units and regional transport systems in hospitals at all levels in China, as well as the popularization of mechanical ventilation and pulmonary surfactant therapy, so the survival rate of premature infants has increased significantly, but the structure and function of gastrointestinal tract in premature infants are immature, which has low level of digestive enzymes, poor digestion and absorption of macromolecular substances, such as protein and fat, and other related gastrointestinal symptoms, such as prone to vomiting, abdominal distension, diarrhea and even bloody stools, and improving the nutritional status of premature infants, which has positive significance to physical and intellectual development in preterm infants and reduce complications. Therefore, the study of nutritional support therapy for premature infants has gradually attracted the attention of clinical medical workers.^[1–4]^ The main goal of nutrition for premature infants is to make the extrauterine growth rate consistent with the intrauterine growth rate of the corresponding gestational age, and the supply of nutrients can meet the needs of the body's tissues and organs. However, premature infants, especially infants with very low or ultra-low birth weight, which are prone to have a slower growth rate after birth than the late intrauterine pregnancy due to gastrointestinal dysfunction, feeding intolerance, and other diseases. And it is the restriction of extrauterine growth and development.^[5–7]^ The smaller the newborn's gestational age and birth weight, the higher the incidence of the restriction of extrauterine growth and development, which will affect the long-term nervous system development. Breast milk is the best food for premature infants, but mothers of premature infants have delayed lactation and the establishment of a breast milk bank in our country is still in its infancy. The lack of a sound management and supervision mechanism is far from meeting the needs of children. When breastfeeding is not possible, it is particularly important to choose suitable formula milk (standard preterm infant formula [SPIF], hydrolyzed protein formula milk, and so on), and improve feeding tolerance.^[8–10]^

Hydrolyzed protein formula milk is processed by heating, ultrafiltration, hydrolysis, etc., which converts large molecules of milk protein into small molecules of short peptides or even free amino acids. The hydrolyzed protein destroys the spatial conformation and changes the epitope. It reduces the antigenicity and is recommended for clinical therapy of children with moderate/severe milk protein allergy and preventive therapy of children with high risk of allergy.^[11,12]^ However, the application of hydrolyzed protein formula is not limited to allergic diseases. Some clinical studies have found that compared with SPIF, hydrolyzed protein formula can speed up gastrointestinal transit, promote gastric emptying, reduce feeding intolerance and the incidence of necrotizing enterocolitis and other gastrointestinal diseases, which is more easily accepted by the immature gastrointestinal tract of newborns, and quickly establishing the total enteral feeding, reducing the risk of adverse reactions, such as infections caused by prolonged parenteral nutrition. In view of the above advantages, there are opinions that hydrolyzed protein formula milk can be used as the first choice for breast milk instead of formula milk.^[13,14]^ However, some studies hold the opposite view, they think that hydrolyzed protein formula cannot reduce the incidence of feeding intolerance and necrotizing enterocolitis. Hydrolyzed protein formula milk is expensive and the smell and taste are unacceptable, which can lead to reduced intake and insufficient feeding, the osmotic pressure of the hydrolyzed formula milk has increased, and the hypertonic fluid passes through the gastrointestinal tract can increase the risk of neonatal necrotizing enterocolitis. In addition, the short peptides produced by artificial hydrolysis may have potential risks to the gastrointestinal tract and it will affect the absorption of nutrients.^[15]^ At present, there are some controversies about the application of hydrolyzed protein formula milk and SPIF in nutritional support therapy of premature infants, this study conducts a meta-analysis on the published clinical randomized controlled trials of preventive hydrolyzed protein formula milk for newborns. To the newborns who cannot breastfeeding, it will adopt a scientific evaluation system to compare the effectiveness and safety of preventive use of hydrolyzed protein formula milk and SPIF in the nutritional support therapy of premature infants, and the effectiveness and safety of preventive use of hydrolyzed protein formula milk in reducing gastrointestinal diseases and promoting physical development of premature infants.

## 2 Methods

This protocol of systematic review and meta-analysis has been registered on open science framework. Registration number: DOI 10.17605/OSF.IO/UQD92. Website link: https://osf.io/uqd92.

### 2.1 Selection criteria

#### 2.1.1 Types of studies

A randomized controlled study comparing the effectiveness and safety of preventive use of hydrolyzed protein formula milk and SPIF in nutritional support therapy of premature infants, language range (Chinese and English). The intervention time of randomized controlled trial to evaluate the effect of hydrolyzed protein formula milk on gastrointestinal diseases was at least 1 week, and that of randomized controlled trial to evaluate the effect on physical development was at least 2 weeks.

#### 2.1.2 Types of patients

All premature low birth weight infants who meet the inclusion and exclusion criteria and require nutritional support therapy.^[16]^

#### 2.1.3 Inclusion criteria

(1) Gestational age≤37 weeks and birth weight <1500 g;
(2) Within 12 hour to 24 hour of birth;
(3) No intrauterine infection, genetic metabolic disease or congenital malformation;
(4) Stable vital signs at admission.

#### 2.1.4 Exclusion criteria

One of the following criteria was excluded:

(1) Newborns with necrotizing enterocolitis, food protein allergic enteritis, feeding intolerance and other related gastrointestinal symptoms before inclusion;
(2) Newborns with congenital gastrointestinal malformations, genetic metabolic diseases and other serious congenital abnormalities;
(3) Newborns with mixed feeding (breast milk combined formula milk);
(4) Using drugs related to gastrointestinal peristalsis and gastric acid secretion;
(5) The first-degree relatives of newborns with allergic diseases;
(6) Animal experiments and case reports, reviews, meeting minutes and other non-randomized controlled trials;
(7) No major outcome indicators required for this study and no literature with relevant raw data available.

#### 2.1.5 Types of interventions

##### 2.1.5.1 Experimental group

Feeding with hydrolyzed protein formula milk.

##### 2.1.5.2 Control group

Feeding with SPIF with the same protein source and similar protein content and energy (the protein source of formula milk is milk protein).

#### 2.1.6 Types of outcome measurements

##### 2.1.6.1 Primary outcome indicators

(1) Time to regain the birth weight;
(2) The incidence of feeding intolerance;
(3) Time required to achieve total enteral feeding;
(4) Physical development (weight, head circumference, body length, body mass growth rate) within 21 days of birth;
(5) The weight gain rate of average daily.

#### 2.1.7 Secondary outcomes

(1) Fetal excretion time;
(2) Frequency of spontaneous defecation;
(3) Extrauterine growth retardation;
(4) Motilin level;
(5) Average formula intake (mL/kg/d);
(6) The incidence of adverse reactions;
(7) Average length of hospital stay.

### 2.2 Literature sources and search

Using computer to search the Chinese database and English database: SinoMed, CNKI, WanFang Data, VIP, PubMed, EMbase and The Cochrane Library, and to collect randomized controlled trials on the application of hydrolyzed protein formula milk in nutritional support treatment of premature infants compared with SPIF. The retrieval time limit is from the establishment of each database to September 1, 2020. The retrieval method of combining subject words and free words is adopted. The logical relationship between subject words is “AND”, and the logical relationship between subject words and free words is “OR”. Searching strategy is shown in Table 1.

**Table 1:** The results are obtained by searching the database (PubMed).

Database	Number	Search items
PubMed	1	((Infant, Newborn) OR (Infant, Premature) OR (Infant, Extremely Premature) OR (Infant, Extremely Low Birth Weight) [Mesh])
	2	((Infant, Newborn) OR (Infants, Newborn) OR (Newborn Infant) OR (Newborn Infants) OR (Newborns) OR (Newborn) OR (Neonate) OR (Neonates) OR (Infant, Premature) OR (Infants, Premature) OR (Premature Infant) OR (Preterm Infants) OR (Infant, Preterm) OR (Infants, Preterm) OR (Preterm Infant) OR (Premature Infants) OR (Neonatal Prematurity) OR (Prematurity, Neonatal) OR (Infant, Extremely Premature) OR (Extremely Premature Infant) OR (Infants, Extremely Premature) OR (Premature Infant, Extremely) OR (Premature Infants, Extremely) OR (Extremely Preterm Infants) OR (Extremely Preterm Infant) OR (Infant, Extremely Preterm) OR (Infants, Extremely Preterm) OR (Preterm Infant, Extremely) OR (Extremely Premature Infants) OR (Infant, Extremely Low Birth Weight) OR (Extremely Low Birth Weight Infant) [Title/Abstract])
	3	((Proteolysis) OR (Protein Hydrolysates) [Mesh])
	4	((Extensively Hydrolyzed Protein Formula) OR (Standard Preterm Infant Formula) OR (Hydrolyzed Protein) OR (Hydrolyzed Casein) OR (Hydrolyzed Whey Protein) OR (Proteolysis) OR (Protein Degradation) OR (Degradation, Protein) OR (Degradations, Protein[Title/Abstract]) OR (Protein Degradations) OR (Protein Digestion) OR (Digestion, Protein) OR (Digestions, Protein) OR (Protein Digestions) OR (Protein Hydrolysates) OR (Hydrolysates, Protein) [Title/Abstract])
	5	((Gastrointestinal Diseases) OR (Child Development) [Mesh])
	6	((Gastrointestinal Diseases) OR (Disease, Gastrointestinal) OR (Diseases, Gastrointestinal) OR (Gastrointestinal Disease) OR (Gastrointestinal Disorders) OR (Gastrointestinal Disorder) OR (Functional Gastrointestinal Disorders) OR (Functional Gastrointestinal Disorder) OR (Gastrointestinal Disorder, Functional) OR (Gastrointestinal Disorders, Functional) OR (Cholera Infantum) OR (Child Development) OR (Development, Child) OR (Infant Development) OR (Development, Infant) [Title/Abstract])
	7	1 OR 2
	8	3 OR 4
	9	5 OR 6
	10	7 AND 8 AND 9

Mesh = medical subject headings.

### 2.3 Data extraction and management

Two authors independently completed the paper search. Deleting the duplicate literature according to the title and abstract, and excluding the literature that obviously does not meet the inclusion criteria. Subsequently, downloading the literature that might be included and read it carefully. When the opinion is inconsistent, the 2 researchers again evaluate the quality of the full text and discuss it until the final agreement is reached. If agreement is not reached in the end, the third researcher decides whether to include the literature. The extracted literature features include:

1. Author, provenance, publication time, language, Jadad score of the literature, and so on.
2. General conditions such as total number of subjects, gestational age, birth weight, day age, and so on;
3. Specific details of interventions and implementation process;
4. The main and secondary outcome indicators.

**Figure 1:**
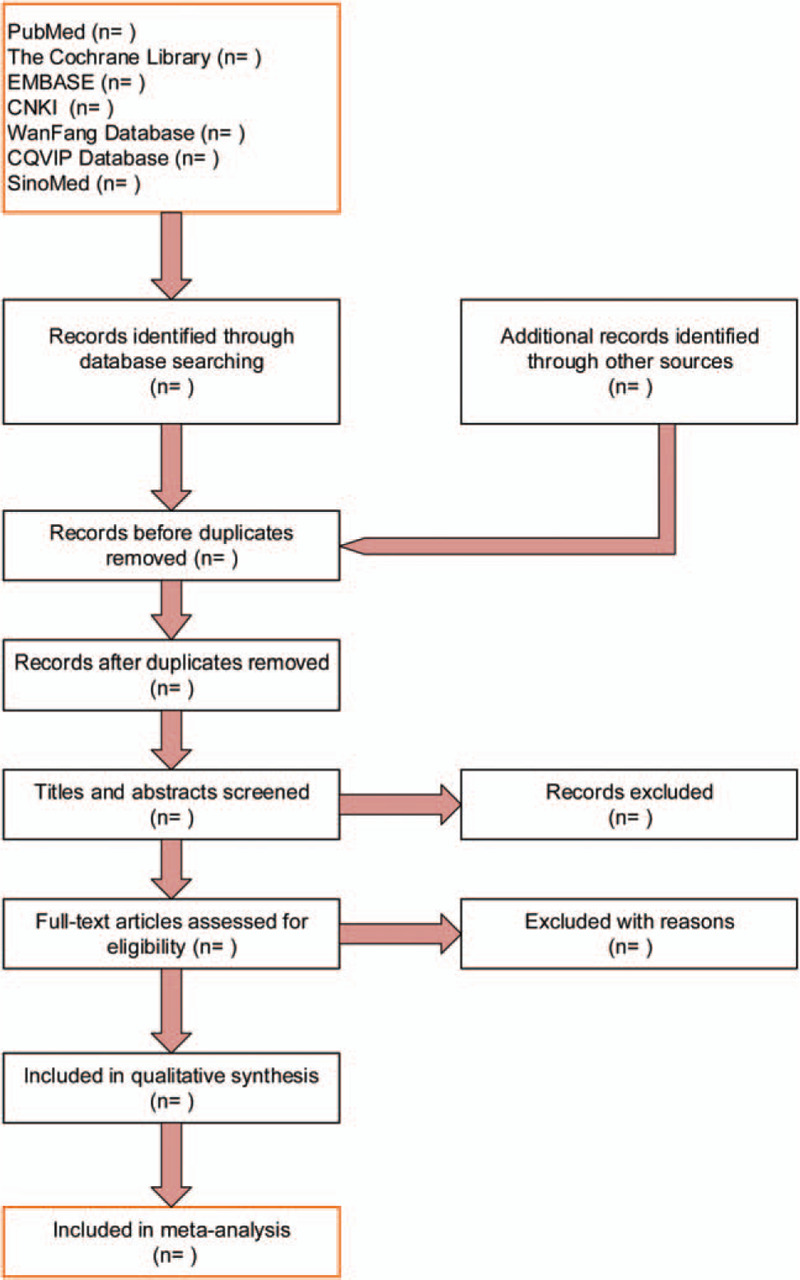
Study selection process for the meta-analysis by PRISMA flow diagram. PRISMA = preferred reporting items for systematic reviews and meta-analyses.

The results of the study selection process are shown in Figure 1.

### 2.4 Literature quality evaluation

The literature quality is evaluated by 2 reviewers using the randomized controlled trial bias risk assessment criteria provided by the Cochrane Collaboration Network and the modified Jadad scale.^[17]^ Modified Jadad score: the full score is 7 points, the higher the score, the higher the quality. The total score is 4 to 7 points is high-quality research, and 1–3 points is low-quality research.

### 2.5 Data analysis

#### 2.5.1 Measures of treatment effect

Using RevMan 5.3.0 to conduct Meta-analysis, and the test results of combined effect size are displayed as forest plots, and using the 95% confidence interval to calculate the odds ratio of binary variables and the mean difference of continuous variables. The P value is used to judge whether the combined effect statistics of multiple studies are statistically significant. Taking *P*<.05 as the difference is statistically significant, and selecting the effect model according to the results of the heterogeneity test. The heterogeneity is measured by I^2^ value and Chi-square test evaluation. If *P* ≤.10 and *I*^2^>50% are statistically heterogeneous between the studies, the random effects model shall be used. If the test result is *P*>.10 and *I*^2^≤50% is no statistical heterogeneity between the studies, then the fixed effects model shall be used. Analyzing the sources of heterogeneity often includes different design schemes, differences in sample size, quality of included studies, and differences in characteristics of research objects.

#### 2.5.2 Subgroup analysis and sensitivity analysis

In this study, the heterogeneity of the indicators will be analyzed by the following factors to reduce the heterogeneity of the results. Such as: the sex of the infants, gestational age, birth weight, hospital stay, and so on.

Sensitivity analysis is to evaluate the influence of each test on the main results and the stability of the results by excluding each study at a time. If the results of sensitivity analysis have not changed greatly, the sensitivity is low and the results are stable and reliable. If the results of sensitivity analysis change greatly, the sensitivity is high and the stability of the results is low, it indicates that there are potential and important bias factors in the study.

### 2.6 Publication Bias

Using the RevMan 5.3.0 software to draw a funnel chart to test whether there is publication bias. When there is publication bias, using STATA 13.0 software to conduct Egger's test, and the funnel map is quantitatively evaluated to determine whether there is publication bias. At the same time, setting *P* < .1 is statistically significant, that is, there is publication bias. When the Egger test reached *P* value <.1, it indicates that there is a publication bias in the outcome indicator, and the sensitivity analysis should be conducted through the clipping method to ensure the robustness of the conclusion.

### 2.7 Ethics

This study belongs to literature analysis and does not need to be reviewed and agreed by the ethics committee.

### 2.8 Evidence evaluation

GRADE was evaluated by GRADE Profiler 3.6, which was analyzed from 5 aspects: limitation, inconsistency, indirectness, accuracy and publication bias. The level of evidence quality was expressed as “high,” “medium,” “low” and “very low.”

## 3 Discussion

In recent years, the incidence of premature infants has increased year by year, and the rescue survival rate of very low and ultra-low birth weight infants has increased significantly. The problems of feeding determine the length of hospital stay and the key to quality of life of premature infants. How to solve the problems of feeding and nutrition has become 1 of the key and hot issues in clinical management of very low birth weight infants.^[18,19]^ Because of the low secretion ability of gastric acid and low digestive enzyme activity of intestinal in premature infants, it affects its hydrolysis of protein and is prone to feeding intolerance, including vomiting, gastric retention and abdominal distension. Early enteral feeding, which can provide enteral nutrition, promote gastrointestinal maturation, increase intestinal enzyme activity and promote postnatal gastrointestinal motility, and it has become the key to determine the quality of life of very low birth weight infants.^[20,21]^ It has been established that breastfeeding in premature infants reduces the risk of feeding intolerance than formula feeding, but the mothers of premature infants have their own diseases or complications during pregnancy, in this case, premature infants often can not achieve breastfeeding, so it often give premature infant formula milk feeding for nutritional support.

As a supplement and substitute for breastfeeding, infant formula milk has been widely used in clinics. According to whether protein is hydrolyzed, infant formula can be divided into SPIF and hydrolyzed protein formula milk. Hydrolyzed protein formula milk is processed to convert macromolecular protein into small molecule short peptide and free amino acid. According to the degree of hydrolysis, it can be divided into partial (moderate) hydrolytic protein formula milk, deep (extensive) hydrolytic protein formula milk, amino acid formula milk. In theory, it is more suitable for newborns than SPIF. Especially, the immature gastrointestinal tract of premature infants, it can promote gastric emptying, and prone to be tolerated, and establish total enteral feeding more quickly. At present, the reason why deep hydrolyzed protein formula milk can improve feeding intolerance is not complete. Clearly, it may be related to the following factors:^[22]^

(1) deep hydrolyzed protein is composed of 80% short peptides + 20% amino acids, which reduces pepsin and intestinal peptidase digestion;
(2) deep hydrolyzed protein formula milk has a small molecular weight, and it may be more conducive to absorption;
(3) deep hydrolyzed protein formula milk can increase motilin levels, promote gastrointestinal motility, and accelerate gastrointestinal emptying;
(4) deep hydrolyzed protein formula milk can reduce intestinal osmotic pressure and accelerate gastrointestinal transit;
(5) deep hydrolyzed protein formula milk reduces the delay in gastrointestinal emptying caused by the release of opioid receptor agonists in the process of casein being digested and absorbed.

At present, the effectiveness of preventive use of hydrolyzed protein formula milk in reducing gastrointestinal diseases is still controversial, and whether hydrolyzed protein formula milk can ensure the normal physical development of newborns needs further verification. Therefore, the effectiveness and safety of preventive use of hydrolyzed protein formula milk are systematically evaluated, and it will give a positive conclusion through this study.

## Author contributions

**Conceptualization:** Qiyan Yang, Qun Lin, Shuli Han.

**Data curation:** Qiyan Yang, Qun Lin, Keni Chen, Juan Cao.

**Funding acquisition:** Shuli Han.

**Investigation:** Qiyan Yang, Qun Lin, Shuli Han.

**Resources:** Qun Lin, Keni Chen, Yonghong Feng.

**Software:** Qiyan Yang, Qun Lin.

**Supervision:** Qiyan Yang, Shuli Han.

**Writing – original draft:** Qiyan Yang, Qun Lin, Keni Chen, Juan Cao, Yonghong Feng.

**Writing – review & editing:** Shuli Han.
